# Circulation of the low pathogenic avian influenza subtype H5N2 virus in ducks at a live bird market in Ibadan, Nigeria

**DOI:** 10.1186/2049-9957-3-38

**Published:** 2014-11-03

**Authors:** Temitope Coker, Clement Meseko, Georgina Odaibo, David Olaleye

**Affiliations:** Virology Department, College of Medicine, University of Ibadan, Ibadan, Nigeria

**Keywords:** LPAI H5N2, Domestic ducks, Live bird market, Circulation, Nigeria

## Abstract

**Background:**

To monitor carrier hosts of avian influenza in Nigeria, we randomly collected cloaca swab specimens from 155 ducks at a live bird market (LBM) in Ibadan, southwest Nigeria, between July 2011 and July 2012.

**Methods:**

The samples were analyzed by real-time reverse transcription-polymerase chain reaction (RT-PCR) and virus isolation was carried out in embryonated chicken eggs. Partial sequencing of the antigenic cleavage site of the haemagglutinin (HA) gene was performed, multiple sequence alignment was carried out using ClustalW, and a phylogenetic tree was constructed using the neighbor joining method.

**Results:**

Twenty (13%) of the 155 samples were positive for avian influenza subtype H5N2 by real-time RT-PCR and three isolates were obtained from embryonated chicken eggs. Partial sequencing of the amino acid cleavage site of the HA genes of two isolates corresponded to a PQRETGL*F sequence that is common in low pathogenic avian influenza (LPAI). Phylogenetically, the HA genes of the two influenza viruses are monophyletic and clustered with H5N2 viruses detected in wild ducks from Africa.

**Conclusion:**

The occurrence of LPAI in domestic ducks in Nigeria underscores the importance of continuous surveillance and monitoring of the virus (in a country that is considered to be free of avian influenza) in order to prevent the emergence of virulent strains that may spread to commercial poultry and humans.

**Electronic supplementary material:**

The online version of this article (doi:10.1186/2049-9957-3-38) contains supplementary material, which is available to authorized users.

## Multilingual abstracts

Please see Additional file 1 for translations of the abstract into the six official working languages of the United Nations.

## Background

The last reported occurrence of highly pathogenic avian influenza (HPAI) H5N1 in Nigeria was in 2008. The virus was detected in asymptomatic ducks at a live bird market (LBM) in Gombe State, by the National Veterinary Research Institute through active surveillance during the nationwide outbreak of the virus in 2006–2008 [1]. Measures to control the transmission and spread of the virus in Nigeria were apparently successful, after the infected farms were disinfected and abolished. Subsequent active and passive surveillance and laboratory diagnosis did not detect avian influenza [2], which contrasts with the situation in other countries such as Egypt, Indonesia, India, and China [3]. However, Nigeria remains at risk of avian influenza reoccurrence due to the regular migration of wild birds, which come into contact with the rural poultry, and through the local trade in poultry that may lead to contamination of the live bird trade chain in rural areas. This study was carried out at a LBM in Ibadan as part of the influenza surveillance activities of the World Health Organization (WHO) National Influenza Center (NIC) of the University of Ibadan, specifically to monitor the potential virus carriers and reservoirs in the country.

## Methods

### Description of the study area

**Figure 1:**
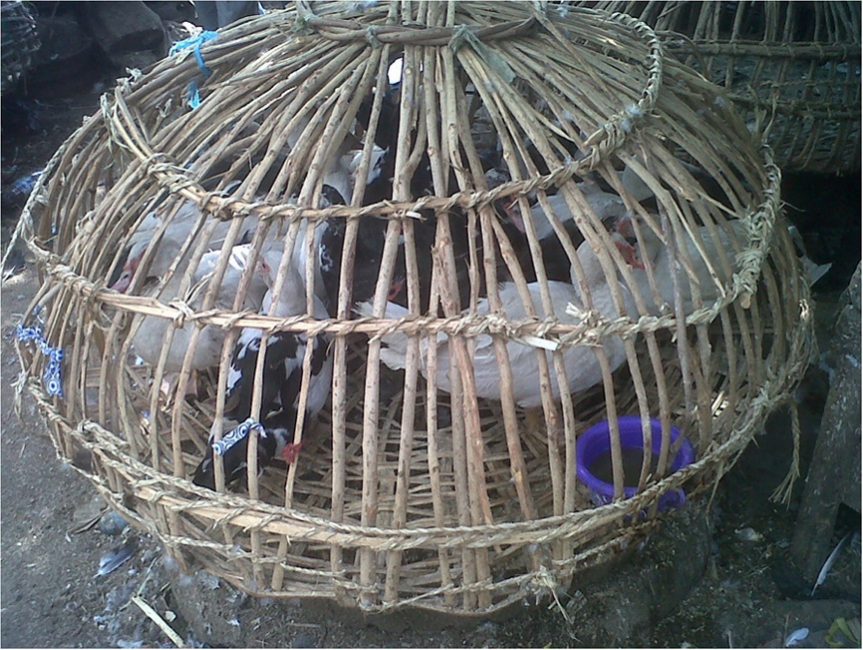
**Ducks in cane baskets at a live bird market, Ibadan, Nigeria.** The birds collected from rural flocks are brought to the market in cane baskets and sold live for slaughter or re-distributed as domestic flock.

The Shasha live bird market (LBM) in Ibadan, southwest Nigeria, was chosen for this study because it serves as a distribution point for free range and rural/backyard birds such as ducks, guinea fowls, pigeons, and chickens. These birds are brought—mainly from rural areas in the northern parts of the country—in cane baskets under poor biosecurity and hygiene (see Figure 1). The market serves as one of the sentinel sites for influenza virus surveillance in southwest Nigeria.

### Sample collection

Cross-sections of subset of ducks brought to the LBM were randomly sampled for this study over a period of one year from July 2011 to July 2012. Ducks were selected over other birds as previous reports showed that they could be infected with avian influenza without apparent clinical signs [4, 5]. Cloaca swab samples were preferred over tracheal swabs when sampling waterfowl [6], and were collected from the ducks by inserting a polyester tipped swab stick in the cloaca and gently twirling it to remove fecal materials with desquamated mucosa cells. These cloaca swabs were then inserted into a commercially prepared viral transport medium (Copan Diagnostics, CA, USA) that included antimicrobial to inhibit bacterial and fungal floral in a clinical specimen. Cloaca swabs containing fecal materials in the viral transport medium were then transported on ice to the laboratory for analysis.

### Nucleic acid extraction and RT-PCR

Fecal materials were homogenized by vortexing and centrifuging to obtain a clear supernatant that was used for ribonucleic acid (RNA) extraction. A Qiagen RNA mini kit (Hilden, Germany) was used to carry out the RNA extraction. This was followed by one-step real-time reverse transcription-polymerase chain reaction (RT-PCR). Primers and fluorescence probes were obtained from the WHO Influenza Collaborating Laboratory, Center for Disease Control (CDC) in Atlanta, USA. The system combines superscript III reverse transcriptase (RT) and platinum Taq DNA Polymerase in a single enzyme mix: both cDNA synthesis and PCR are performed in a single reaction. Gene specific primers and probes designed for the generic matrix gene (F: 5′-AGATGAGTCTTCTAACCGAGGTCG-3′ R: 5′-TGCAAAGACACTTTCCAGTCTCTG-3′, FAM- 5′- TCAGGCCCCCTCAAAGCCGA-3′) were used. The master mix was prepared by dispensing 5.5 ul nuclease free water; 0.5 ul of forward, reverse primers, and probe; 0.5 ul superscript Tag polymerase; and 12.5 ul PCR master mix enzyme (2×) for a single reaction multiplied by the number of samples tested. The Applied Biosystems fast real-time PCR system 7,500 thermocycler, and software (Applied Biosystems, Foster City, CA, USA) were used with the following cycling conditions: 50°C for 30 minutes, 95°C for 15 minutes, followed by 40 cycles of 95°C for 10 seconds, and 60°C for 30 seconds and 5–60 seconds [7]. Samples that were positive for the M-gene, which codes for the matrix protein common to all influenza A viruses, were subtyped under the same conditions with H5 (F: 5′- TTATTCAACAGTGGCGAG-3′, R: 5′- CCAKAAAGATAGACCAGC-3′, P: 5′- CCCTAGCACTGGCAATCATG-3′) and H1 (F: 5′-GTG CTA TAA ACA CCA GCC TYC CA-3′, R: 5′-CGG GAT ATT CCT TAA TCC TGT RGC 3′, P: 5′- CA GAA TAT ACA “T”CC RGT CAC AAT TGG ARA A 3′).

### Virus isolation and identification

Virus isolation was performed by inoculating nine-day old embryonating eggs with a supernatant fluid of cloaca swab specimens. Dead embryo post inoculations were chilled, the harvested egg supernatant fluid was analyzed by haemagglutination, and haemagglutination inhibition was performed according to the WHO protocols. A haemagglutination test was carried out using a V-bottom microtiter plate. The test entailed a two-fold serial dilution of the virus suspended in phosphate buffered saline and a 1% suspension of chicken red blood cells (RBC) added and incubated at 25°C for 30 minutes, in order to determine the haemagglutination titer of the virus. Thereafter, haemagglutination inhibition (HI) was carried out using H5, H3, and H1 reference antisera obtained from the Food and Agriculture Organization (FAO)/World Organization for Animal Health (OIE) Reference Center for Animal Influenza and Newcastle Disease Virus, Istituto Zooprofilattico Sperimentale delle Venezie, Padua, Italy, and the WHO/CDC influenza surveillance kit for 2011. Two fold serial dilution of reference antisera in phosphate buffered saline was performed in a V-bottom microtiter plate after which predetermined 4HA units of test virus were added and allowed to incubate at 25°C for 30 minutes. Chicken RBC suspension (1%) was added and re-incubated for another 30 minutes. The HI titer was thereafter determined using a microtiter plate reader or by observing teardrop streaming of the microtiter plate when tilted. This indicates alpha haemagglutination inhibition by the virus against a homologous reference antiserum.

### Gene sequencing and phylogeny

Following virus isolation and identification, partial sequencing of the antigenic cleavage site of the haemagglutinin (HA) gene was performed to identify molecular determinants of avian influenza virulence, which are monobasic amino acids adjacent to the cleavage site of the HA [8]. The HA segment of two avian influenza isolates were sequenced by the Sanger method using BigDye^®^ Terminator v3.1 (Applied Biosystems, Foster City, CA) and phylogenetically analyzed along with selected influenza A/H5 sequences available in GenBank. The Basic Local Alignment Search Tool (BLAST) was initiated in the National Council for Biotechnology Information (NCBI) platform (http://www.ncbi.nlm.nih.gov) to obtain sequences that are similar to our isolates. Other H5 sequences from ducks in Nigeria and Africa were also included and sequence data were compiled and edited using the MEGA 5 bioinformatic software (http://www.megasoftware.net). Multiple sequence alignment was carried out using ClustalW and a phylogenetic tree was constructed using the neighbor joining method. Bootstrap values were calculated on 1,000 replicates of the alignment. Gene sequencing and neuraminidase sub-typing against a panel of N1 to N9 primers was carried out at the OIE Reference Center for Animal Influenza and Newcastle Disease Virus, in Padua, Italy.

## Results

### Study site

The live bird market (LBM) occupies a section of a general commodity market in a suburban area of the city. Traders deal in various species of birds including ducks, chickens, guinea fowls, geese, and pigeons. Birds were separated in cages by species and age. Officials of the Ministry of Agriculture perform occasional disinfestations at the market as part of the national program to control avian influenza.

### Molecular detection, virus isolation, and subtyping

**Figure 2:**
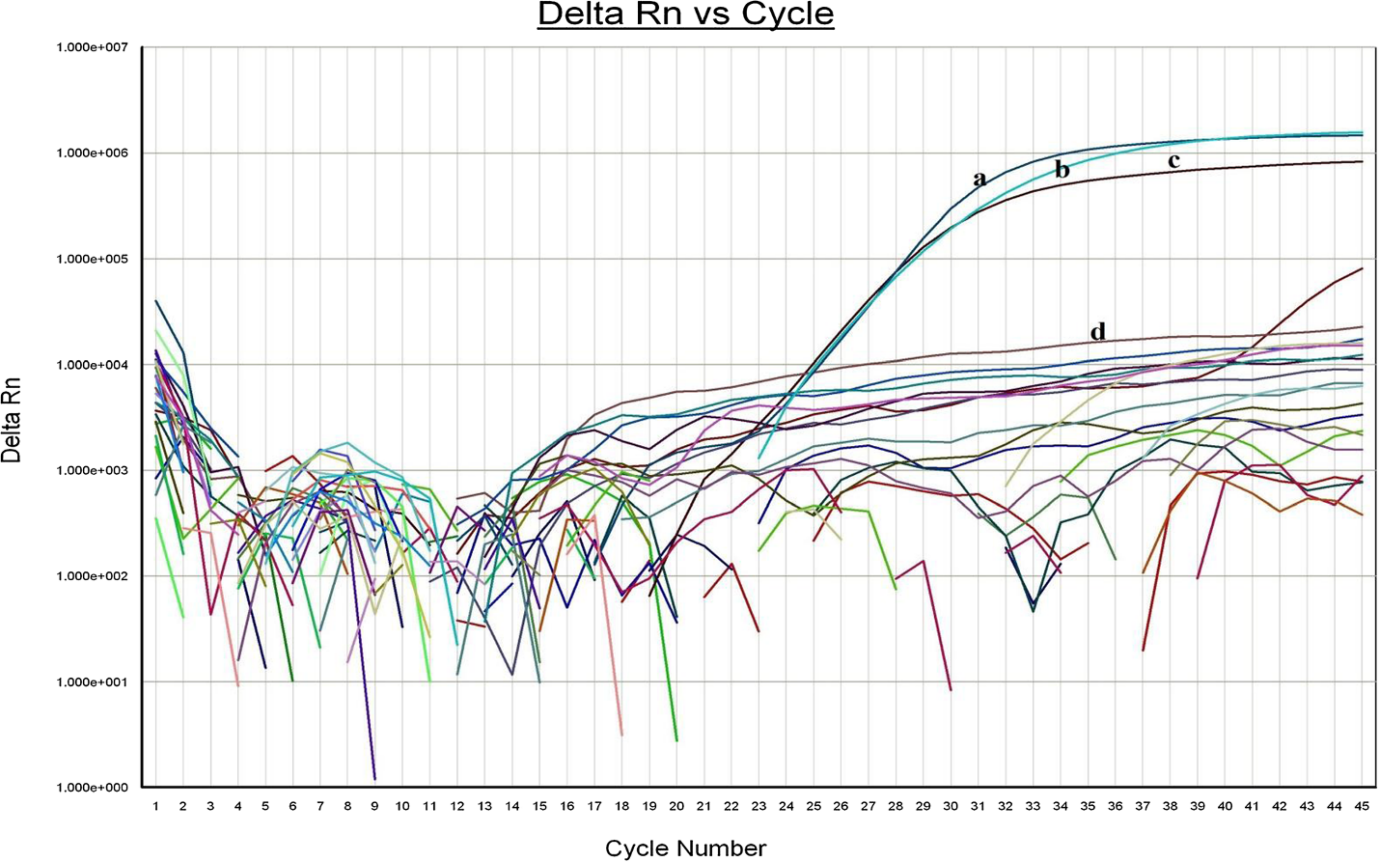
**RT-PCR curve of influenza A/H5 subtype.** The RT-PCR curve of influenza A/H5 subtyping: (a-positive control, c, and b-positive samples all showing exponential rise in the graph; d-negative control and all other lines without exponential rise were negative). The cycle threshold (CT) for both positive control and positive samples is 25 as shown in the x-axis.

Twenty (13%) out of the 155 randomly collected cloaca swab samples in ducks from the LBM (see Additional file 2) were positive for avian influenza virus subtype H5 by real-time reverse transcription-polymerase chain reaction (RT-PCR) (see Figure 2). The real-time RT-PCR cycle threshold (CT) was ≥25 in all of the positive specimens. The positive samples were collected between September 2011 and February 2012, a time of year which corresponds to the cold, dry, and dusty harmattan season in Sub-Saharan Africa. This kind of climate favors persistence of respiratory pathogens such as avian influenza. Three isolates were obtained from embryonated eggs with a haemagglutinin (HA) titer of >12log^2^ on second passage. The isolates were inhibited by reference H5 antiserum, and were negative for the H1 and H3 influenza subtypes tested for. Neuraminidase sub-typing of the isolates was positive for N2 and negative for other subtypes.

### Nucleotide sequencing and phylogenetic analysis

**Figure 3:**
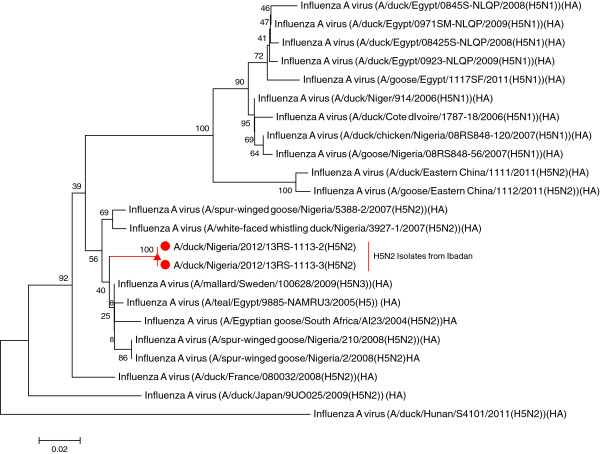
**Phylogenetic tree of the HA gene of LPAI H5N2 virus from Ibadan, Nigeria.** Phylogenetically, the HA gene of the two LPAI H5N2 domestic duck isolates from Ibadan, Nigeria are monophyletic (100% homologous indicated at the node) and clustered with other H5N2 isolates from wild ducks in Africa and Europe.

Two of the three isolates were sequenced and the amino acid sequence at the cleavage site of the HA corresponded to PQRETGL*F, which is typical of low pathogenic avian influenza (LPAI). Compared with LPAI H5N2 strains previously detected in white-faced whistling ducks and spur-winged geese in Nigeria, the LPAI H5N2 isolated in domestic ducks showed amino acid substitutions N289D, K341T, and I364V, which are not as important as the predicted amino acid sequence of the cleavage site which showed no marker for virulence. Phylogenetically, the HA genes of the two influenza viruses are monophyletic (100% homologous indicated at the nodes) and generally clustered with LPAI H5N2 strains earlier isolated in wild ducks in Europe and Africa (see Figure 3). However, A/duck/Nigeria/2012/13RS-1113-2 and 3 (H5N2) formed a distinct cluster away from A/spur-winged goose/Nigeria/5388-2/2007 (H5N2), and A/white-faced whistling duck/Nigeria/5388-2 (H5N2) that were detected in 2007, which also showed different homology from the 2008 isolates. All LPAI H5N2 strains from Nigeria appeared to have originated from viruses from mallard and wild ducks from Sweden and France, respectively. The obtained partial amino acid sequence was deposited into GenBank with the accession number KJ917546 [9].

## Discussion

Ducks play a significant role in the maintenance and transmission of highly pathogenic avian influenza (HPAI) globally [10]. Several cases of successful isolations of HPAI virus from domestic birds, including ducks, at a live bird market (LBM) were reported in Nigeria between 2006 and 2008 [11]. The last confirmed case of HPAI in Nigeria was detected in a duck at a LBM during active surveillance for the influenza virus [1]. Low pathogenic avian influenza (LPAI) H5N2 was also reported in a wild bird in 2008, but the virus was not maintained in the environment [12]. The detection of circulating LPAI H5N2 in domestic waterfowl, four years after avian influenza virus was considered eradicated from the country, is significant in many ways. Wild and domestic ducks have been speculated to be an important link in the transmission of the avian influenza virus between migratory birds and domestic chickens [12, 13]. Free range domestic ducks in rural areas come into contact with wild ducks and can harbor the virus without showing any clinical signs [6]. Hence, an apparently healthy duck that is mixed with other birds at a LBM with poor hygiene and biosecurity measures, as shown in this study, could easily transmit the avian influenza virus to other birds and/or humans.

In a study by Snoeck *et al.* [14], LPAI H5N2 was detected in African wild birds, specifically in the Hadejia-Nguru wetlands in northern Nigeria in 2008. According to the authors, infected wild birds represent a risk for poultry as they may introduce LPAI viruses into free ranging domestic ducks rearing in the wetland areas in northeast Nigeria, with whom they occasionally mix. This has been confirmed by our study as most of the domestic ducks sold at the LBM, where our study was carried out, were brought from northern Nigeria. Spur-winged geese or whistling ducks which were previously positive for LPAI H5N2 in the wild [12, 14] may have transmitted H5N2 to domestic ducks and domestic chickens. Human handlers working at the LBM can also be infected.

Since 2003, HPAI has remained endemic in southeast Asia and North Africa (Egypt). In 2012 alone, there were several outbreaks in poultry farms and LBMs and about 20 human cases of which 15 were lethal [15] *.* ProMED disease alerts also showed preponderance of H5N1 in ducks at LBMs in New York, Germany, Mexico, and China [15]. In June 2013, the World Organization for Animal Health (OIE) was notified of an incidental finding of a subclinical infection with LPAI serotype H5 in a duck in a backyard flock in Australia [16]. In Nigeria, however, there have been no reported cases of either HPAI or LPAI infection in domestic poultry since 2008 [2]. This has given the impression that Nigeria was free from avian influenza, resulting in the OIE declaring it disease free in 2013 [17]. The results of this study, however, showed that the avian influenza virus is circulating in a less apparent form and in less susceptible species such as domestic ducks.

Since March 2013, sporadic transmission of avian influenza H7N9, not previously known in humans, was reported in several fatal cases of avian influenza infection in China [17]. Though the Asian H7N9 virus is a low pathogenic strain in poultry, the clinical outcome of infection in humans is severe. Genetic changes in amino acid sequences have been associated with adaptations leading to enhanced virus binding to, and replication in, mammalian respiratory cells with increased severity of infections [18]. Reversal to virulence in birds has also been documented in the past, where a LPAI strain became highly pathogenic. This was observed with H5N2 in Pennsylvania (1983), in Mexico (1994), and with H7N1/H7N3 in Italy (1997) and Canada (2002) [19], and is not impossible in Nigeria. This is particularly the case as the Nigerian LPAI H5N2 strains belong to a genetic cluster that seems to have an increased propensity to develop the highly pathogenic phenotype [14]. Continuing avian influenza virus surveillance is therefore important for monitoring these genetic changes and biological host adaptations in countries that are most at risk, especially those with a history of outbreaks. Animal sampling should be emphasized to ascertain the extent of the reservoir of avian influenza H5 or H7 viruses especially at LBMs. This study therefore serves as an early warning that avian influenza, albeit a low pathogenic strain, circulates in Nigeria.

## Conclusion

This report shows the importance of continuous surveillance of the avian influenza virus especially in geographical areas with a history of outbreaks. The study also emphasizes the need to include potential carriers and reservoirs of the virus in surveillance programs in order to prevent future outbreaks in commercial poultry and human populations in Nigeria.

## Electronic supplementary material

Additional file 1: **Multilingual abstracts in the six official working languages of the United Nations.** (PDF 314 KB)

Additional file 2: **Sample distribution of cloaca swab specimens collected from ducks between July 2011 and July 2012.** Distribution of cloaca swab specimens collected from ducks between July 2011 and July 2012 by date, sex, age and results of laboratory analyses. (XLSX 14 KB)

## Abbreviations

CDC: Center for disease control
CT: Cycle threshold
HA: Haemagglutinin or Haemagglutination
HI: Haemagglutination inhibition
HPAI: Highly pathogenic avian influenza
LBM: Live bird market
LPAI: Low pathogenic avian influenza
NIC: National influenza center
OIE: World Animal Health Organization
RNA: Ribonucleic acid
RT: Reverse transcriptase
RT-PCR: Reverse transcription-polymerase chain reaction
RBC: Red blood cells
WHO: World Health Organization.

## References

[1] Fusaro A, Joannis T, Monne I, Salviato A, Yakubu B, Meseko C, Fassina S, Capua I, Cattoli G (2009). Introduction into Nigeria of a distinct genotype of avian influenza virus (H5N1). Emerg Infects Dis.

[2] Oladokun AT, Meseko CA, Ighodalo E, John B, Ekong PS (2012). Effect of intervention on the control of highly pathogenic avian influenza in Nigeria. Pan Afr Med J.

[3] World Health Organization: **Influenza at the human-animal interface.** http://www.who.int/influenza/human_animal/about/ent Feb. 2012 (Accessed 20^th^ February 2013)

[4] Halvorson D, Karunakaram D, Senne D, Bailey C, Abraham A, Hinshaw V, Newman J (1983). Epizootiology of Avian Influenza- simultaneous monitoring of sentinel ducks and turkeys in Minnesota. Avian Dis.

[5] Meseko CA, Oladokun AT, Shehu B (2007). An outbreak of Highly Pathogenic Avian Influenza (HPAI) in a mixed farm by the introduction of a waterfowl. Niger Vet J.

[6] Ip HS, Dusek RJ, Heisey DM (2012). The effect of swab sample choice on the detection of avian influenza in apparently healthy wild ducks. Avian Dis.

[7] Ducatez MF, Hause B, Stigger-Rosser E, Darnell D, Corzo C, Juleen K, Simonson R, Brockwell-Staats C, Rubrum A, Wang D, Webb A, Crumpton J, Lowe J, Webby RJ (2011). Multiple reassortment between pandemic (H1N1) 2009 and endemic influenza viruses in pigs. Emerg Infect Dis.

[8] Gohrbandt S, Veits J, Hundt J, Bogs J, Breithaupt A, Teifke JP, Weber S, Mettenleiter TC, Stech J (2011). Amino acid adjacent to haemagglutination site are relevant for virulence of avian influenza subtype H5. J Gen Virol.

[9] Bao Y, Bolotov P, Dernovoy D, Kiryutin B, Zaslavsky L, Tatusova T, Ostell J, Lipman D (2008). The influenza virus resource at the national center for biotechnology information. J Virol.

[10] Alexander DJ (2000). A review of avian influenza in different bird species. Vet Microbiol.

[11] Henning J, Bett B, Okike I, Abdu P, Perry B (2012). Incidence of highly pathogenic avian influenza H5N1 in Nigeria, 2005–2008. Transbound Emerg Dis.

[12] Gaidet N, Cattoli G, Hammoumi S, Newman SH, Hagemeijer W, Takekawa JY, Cappelle J, Dodman T, Joannis T, Gil P, Monne I, Fusaro A, Capua I, Manu S, Micheloni P, Ottosson U, Mshelbwala JH (2008). Evidence of infection by H5N2 highly pathogenic avian influenza viruses in healthy wild waterfowl. PLoS Pathog.

[13] World Organization for Animal Healthhttp://www.oie.int/animal-health-in-the-world/update-on-avian-influenza 2012. (Accessed 20^th^ February 2013)

[14] Snoeck CJ, Adeyanju AT, De Landtsheer S, Ottosson U, Manu S, Hagemeijer W, Mundkur T, Muller CP (2011). Reassortant low-pathogenic avian influenza H5N2 viruses in African wild birds. J Virol.

[15] ProMEDMAIL: *International Society of Infectious Diseases*. http://www.promedmail.org (Accessed 10^th^ March 2013)

[16] World Organisation for Animal Health (OIE): *World Animal Health Information Database, Weekly Disease Information 2013; 26 (11)*. http://www.oie.int/wahis_2/public/wahid.php/Diseaseinformation/reportarchive (Accessed 11^th^ July, 2014)

[17] World Health Organization (2013). Global Alert and Response (GAR): Human Infection with Influenza A (H7N9) Virus in China.

[18] Gao R, Cao B, Hu Y, Feng Z, Wang D, Hu W, Chen J, Jie Z, Qiu H, Xu H, Xu X, Lu H, Zhu W, Gao Z, Xiang N, Shen Y, He Z, Gu Y, Zhang Z, Yang Y, Zhao X, Zhou L, Li X, Zou S, Zhang Y, Li X, Yang L, Guo J, Dong J, Li Q (2013). Human infection with a novel avian-origin influenza A (H7N9) virus. N Engl J Med.

[19] Horimoto T, Kawaoka Y (2005). Influenza: lessons from past pandemics, warning from current incidents. Nat Rev Microbiol.

