# Supplementary material for: Circulation of the low pathogenic avian influenza subtype H5N2 virus in ducks at a live bird market in Ibadan, Nigeria
Source: Infect Dis Poverty. 2014 Nov 3;3:38. doi: 10.1186/2049-9957-3-38 (PMC4322551; doi:10.1186/2049-9957-3-38)
Supplement: Supplementary file 1 — Additional file 1: Multilingual abstracts in the six official working languages of the United Nations. (PDF 314 KB) [file 40249_2014_81_MOESM1_ESM.pdf]

Translation of the abstract into the six official working languages of the United Nations

انتشار مرض انفلونزا الطيور منخفض العدوى من سلالة فيروس H5N2 في البط في سوق للطيور الحية في إيبادان، نيجيريا

تيميتوب كوكر وكليمينت ميسيكو وجيورجينا أوديبو وديفيد أولاليي*

الملخص

معلومات أساسية: من أجل مراقبة الطيور الحاملة لأنفلونزا الطيور في نيجيريا، جمعنا عشوائياً عينات مسحة المذرق من 155 بطة في سوق الطيور الحية (LBM) في أبيدجان، جنوب غرب نيجيريا، خلال الفترة من يوليو 2011 وحتى يوليو 2012.

طرق البحث: تم تحليل العينات بواسطة مقايسة المنتسخة العكسية لتفاعل البوليميراز المتسلسل (**RT-PCR**) في الوقت الحقيقي وتم إجراء عزل الفيروس في أجنة بيض الدجاج. تم تنفيذ التسلسل الجزئي لموقع انشقاق الأنتيجين في جين الراصة الدموية (**HA**)، وتم إجراء محاذاة تسلسل متعددة باستخدام برنامج كلوستال دبليو (**ClustalW**)، وتم بناء الشجرة التطورية باستخدام أسلوب التجميع المجاور.

النتائج: عشرون عينة (13٪) من إجمالي 155 عينة كانت ايجابية بإنفلونزا الطيور من سلالة H5N2 بواسطة مقايسة المنتسخة العكسية لتفاعل البوليميراز المتسلسل (**RT-PCR**) في الوقت الحقيقي، وتم كذلك الحصول على ثلاث عزلات من أجنة بيض الدجاج. وتطابق التسلسل الجزئي التسلسل الجزئي لموقع انشقاق الحمض الأميني في جينات الراصة الدموية (**HA**) في اثنين من العزلات مع تسلسل PQRETGL*F والذي يعد شائعاً في مرض انفلونزا الطيور منخفض العدوى (LPAI). وفيما يتعلق بالتطور السلالي، كانت جينات HA لفيروسي الأنفلونزا أحادية النمط الخلوي ومتجمعة مع فيروسات H5N2 التي تم رصدها في طيور البط البري من أفريقيا.

الاستنتاج: يؤكد ظهور مرض انفلونزا الطيور منخفض العدوى (LPAI) في البط المحلية في نيجيريا أهمية المراقبة المستمرة ورصد الفيروس (في بلاد تعتبر خالية من انفلونزا الطيور) وذلك لمنع ظهور سلالات خبيثة قد تنتشر إلى الدواجن التجارية والبشر.

Translated from English version into Arabic by Wahib Farhan, through

TRANSLATORS
WITHOUT BORDERS

尼日利亚伊巴丹活禽交易市场鸭子中 H5N2 低致病性禽流感流行情况调查结果分析

Temitope Coker, Clement Meseko, Georgina Odaibo and David Olaleye*

摘要

引言：为了了解尼日利亚禽流感的流行情况，2011 年 7 月至 2012 年 7 月间，我们在尼日利亚西北部的伊巴丹活禽交易市场随机采集了 155 份鸭泄殖腔试子样本进行禽流感病毒检测。

方法：采用 RT-PCR 对所有样本进行检测并在鸡胚中进行病毒分离，对 HA 基因抗原裂解位点的部分序列进行序列分析，使用 ClustalW 软件和邻接法分别进行多序列比对和进化树的构建分析。

结果：在 155 份样本中共有 20 份样本（13%）经 RT-PCR 检测为 H5N2 禽流感病毒阳性，在鸡胚中分离到 3 株病毒，其中 2 株病毒 HA 基因氨基酸裂解位点序列分析对应为 PQRETGL*F 序列，为低致病性禽流感病毒（LPAI）。系统发育树分析显示，2 株流感病毒 HA 基因是单系群且与从非洲野生鸭子体内检测到的 H5N2 病毒聚集。

结论：在尼日利亚当地鸭子中检测到低致病性禽流感病毒（LPAI），凸显了开展持续性的禽流感病毒调查和监测的重要性。在一个国家内禽流感病毒是自由流动的，开展禽流感病毒的调查和监测，对预防和控制禽流感病毒在经济禽类和人群中蔓延是非常必要的。

Translated from English version into Chinese by Tian Li-guang, through

CDC CHINESE CENTER FOR DISEASE CONTROL AND PREVENTIOI
NATIONAL INSTITUTE OF PARASITIC DISEASES

Détection d'un virus faiblement pathogène de grippe aviaire du sous-type H5N2 chez des canards dans un marché d'oiseaux vivants à Ibadan au Nigeria.

Temitope Coker, Clement Meseko, Georgina Odaibo et David Olaleye*

Description

Contexte : Afin de surveiller les potentiels porteurs de la grippe aviaire au Nigeria, nous avons effectué des prélèvements aléatoires du cloaque sur 155 canards dans un marché d'oiseaux vivants à Ibadan dans le sud ouest du Nigeria entre juillet 2011 et juillet 2012.

Méthodes : Les échantillons ont été analysés par réaction en chaîne à la polymérase quantitative (PCR quantitative) et le virus a été isolé par culture dans des œufs de poules fécondés. Nous avons réalisé un séquençage partiel du site de clivage de l'antigène de l'hémagglutinine (HA) ainsi que l'alignement multiple de séquences avec ClustalW et un arbre phylogénétique a été construit grâce à la méthode neighbour joining.

Résultats : Vingt (13%) des 155 échantillons ont été testés positifs pour la grippe aviaire H5N2 par PCR quantitative et trois isolations du virus ont été obtenues dans les cultures d'œufs de poules fécondés. Le séquençage partiel du site de clivage de l'acide aminé du gène HA de 2 isolations correspond à la séquence PQRETGL*F qui est commune à tous les virus faiblement pathogènes de grippe aviaire (VFPGA). Phylogénétiquement parlant, les gènes HA des deux virus de la grippe sont monophylétiques et associés aux virus H5N2 détectés chez les canards sauvages d'Afrique.

Conclusion : Le nombre de cas de VFPGA chez les canards domestiques au Nigeria montre l'importance d'une surveillance continue et d'un contrôle permanent du virus (dans les pays considérés comme n'hébergeant pas de porteur de la grippe aviaire) afin d'éviter la recrudescence de souches virulentes qui pourraient être transmises aux volailles industrielles voire à l'homme.

Translated from English version into French by Fanny, through

TRANSLATORS
WITHOUT BORDERS

Распространение вируса слабопатогенного птичьего гриппа подтипа H5N2 у уток на рынке живых птиц в городе Ибадан, Нигерия

Темитоп Кокер, Клемент Месеко, Георгина Одайбо и Давид Олалейе (Temitope Coker, Clement Meseko, Georgina Odaibo and David Olaleye*)

Отрывок

История вопроса: Для того чтобы отконтролировать переносчиков птичьего гриппа в Нигерии, мы в произвольном порядке отбирали образцы посредством взятия мазков из клоаки у 155 уток на рынке живых птиц в городе Ибадан, на юго-западе Нигерии, в период с июля 2011 по июль 2012 года.

Методы: Анализ образцов выполнялся посредством метода полимеразной цепной реакции с обратной транскриптазой в реальном времени (RT-PCR), а выделение вируса было произведено в оплодотворенных куриных яйцах. Было произведено частичное секвенирование антигенного сайта расщепления гена гемагглютинина, множественное выравнивание последовательностей было произведено при использовании ClustalW, а филогенетическое древо было построено при помощи метода объединения соседних пар.

Результаты: Двадцать (13%) из 155 образцов дали положительный результат на птичий грипп подтипа H5N2 посредством метода RT-PCR, и из оплодотворенных куриных яиц были получены три штамма. Частичное секвенирование антигенного сайта аминокислоты генов гемагглютинина двух штаммов соответствовало последовательности PQRETGL*F, характерной для слабопатогенного птичьего гриппа. Филогенетически, гены гемагглютинина двух вирусов птичьего гриппа являются монофилетическими и объединены с вирусами H5N2, выявленными у диких уток из Африки.

Заключение: Распространенность слабопатогенного птичьего гриппа в Нигерии демонстрирует важность непрерывного контроля и мониторинга вируса (в стране, которая считается свободной от птичьего гриппа) для предупреждения появления вирулентных штаммов, которые могут распространиться на промышленную птицу и людей.

Translated from English version into Russian by tatiana_com, through

TRANSLATORS WITHOUT BORDERS

Circulación del subtipo H5N2 del virus de la gripe aviar de baja patogenicidad en patos en un mercado de aves vivas situado en Ibadan (Nigeria)

Temitope Coker, Clement Meseko, Georgina Odaibo y David Olaleye*

Resumen

Información de referencia: Con el objetivo de monitorizar a los portadores de la gripe aviar en Nigeria hemos recogido al azar muestras de frotis de cloaca de 155 patos en un mercado de aves vivas situado en Ibadan (sureste de Nigeria), entre julio de 2011 y julio de 2012.

Métodos: Las muestras se analizaron mediante reacción de cadena de la polimerasa con transcripción inversa en tiempo real (RT-PCR) y se llevó a cabo el aislamiento del virus en huevos embrionados. Se obtuvo la secuencia parcial del punto de división antigénica del gen de la hemaglutinina (HA), se realizó la alineación de múltiples secuencias mediante ClustalW y se construyó un árbol filogenético utilizando el método de unión de vecinos.

Resultados: Veinte (13%) de las 155 muestras fueron positivas para el subtipo H5N2 de gripe aviar mediante RT-PCR en tiempo real y se obtuvieron tres aislados de los huevos embrionados. La secuencia parcial del punto de división del aminoácido de los genes de HA de dos aislados corresponden a una secuencia de PQRETGL*F que es común en la gripe aviar de baja patogenicidad. Desde un punto de vista filogenético, los genes de HA de los dos virus de la gripe son monofiléticos y se agrupan a los virus H5N2 detectados en patos salvajes de África.

Conclusión: La incidencia de la gripe aviar de baja patogenicidad en patos domésticos en Nigeria subraya la importancia de la vigilancia continua y la monitorización del virus (en un país que se considera libre de la gripe aviar) con el fin de prevenir la aparición de cepas virulentas que puedan difundirse a las aves de corral y los humanos.

Translated from English version into Spanish by sergiman, through

TRANSLATORS
WITHOUT BORDERS
